# Modification of tobacco plant development by sense and antisense expression of the tomato viroid-induced AGC VIIIa protein kinase PKV suggests involvement in gibberellin signaling

**DOI:** 10.1186/1471-2229-9-108

**Published:** 2009-08-18

**Authors:** Rosemarie W Hammond, Yan Zhao

**Affiliations:** 1 Molecular Plant Pathology Laboratory, United States Department of Agriculture, Agricultural Research Service, Beltsville, Maryland 20705, USA

## Abstract

**Background:**

The serine-threonine protein kinase gene, designated *pkv *(protein kinase- viroid induced) was previously found to be transcriptionally activated in tomato plants infected with the plant pathogen *Potato spindle tuber viroid *(PSTVd). These plants exhibited symptoms of stunting, and abnormal development of leaf, root, and vascular tissues. The encoded protein, PKV, is a novel member of the AGC VIIIa group of signal-transducing protein kinases; however, the role of PKV in plant development is unknown. In this communication, we report the phenotypic results of over expression and silencing of *pkv *in transgenic tobacco.

**Results:**

Over expression of *pkv *in *Nicotiana tabacum *cv. Xanthi (tobacco) resulted in stunting, reduced root formation, and delay in flowering, phenotypes similar to symptoms of PSTVd infection of tomato. In addition, homozygous T2 tobacco plants over expressing PKV were male sterile. Antisense expression of *pkv*, on the other hand, resulted in plants that were taller than non-transformed plants, produced an increased number of flowers, and were fertile. Exogenous application of GA_3 _stimulated stem elongation in the stunted, sense-expressing plants. PKV sense and antisense expression altered transcript levels of GA biosynthetic genes and genes involved in developmental and signaling pathways, but not genes involved in salicylic acid- or jasmonic acid-dependent pathways. Our data provide evidence suggesting that PKV plays an important role in a GA signaling pathway that controls plant height and fertility.

**Conclusion:**

We have found that the over expression of the tomato protein kinase PKV resulted in stunting, modified vascular tissue development, reduced root formation, and male sterility in tobacco, and we propose that PKV regulates plant development by functioning in critical signaling pathways involved in gibberellic acid metabolism.

## Background

Viroids, the smallest known pathogenic agents of plants, are distinguished from plant viruses by their lack of a protein coat and absence of protein coding capacity [1]. Covalently-closed, circular RNA molecules ranging in size from 239-401 nucleotides, viroids replicate and move from cell to cell and throughout the plant without a helper virus, but with the aid of host cell components [2]. *Potato spindle tuber viroid *(PSTVd) and the pospiviroids replicate and accumulate in the nucleus [3,4]. PSTVd causes a serious disease of tomato characterized by stunting, abnormal development of root and vascular tissues, leaf epinasty and deformation; the severity of symptoms ranging from mild to lethal depending on the viroid strain [5]. Many of the symptoms caused by viroid infection suggest an imbalance in growth hormones. A significant decrease in endogenous gibberellins (GA_3 _and/or GA_1_) has been observed in viroid-infected plants [6].

The molecular basis of symptom formation in viroid-infected plants is unknown, although the nuclear location of PSTVd suggests interactions with the host genome and/or transcription machinery. Viroid infection of tomato results in increased transcription of defense-related genes [7-14]. Using macroarray technology, Itaya et al. [15] also observed induction or suppression of genes encoding proteins involved in stress responses, cell wall structure, chloroplast function, and protein metabolism. Growth reduction in citrus caused by infection with *Citrus exocortis viroid *(CEVd) (a pospiviroid) was correlated with reduced levels of gibberellin 20-oxidase (GA20ox) mRNA [16], and Qi and Ding [17] reported that *LeExp2 *expansin gene expression is down-regulated in viroid-infected tomato plants, suggesting that stunting results from restricted cell expansion.

Although gene expression in viroid-infected plants is altered, little is known of the link between the viroid infection process and gene activation or suppression and resulting symptoms. Single or multiple nucleotide substitutions in the pathogenicity region [18-21] or the central conserved region [17] of PSTVd can dramatically alter symptoms in infected tomato plants. Surprisingly, constitutive expression a secondary hairpin derived from the pathogenicity region of the virulent RG1 strain of PSTVd resulted in viroid symptoms in transformed tomato [22]. RNA-induced silencing of host genes was proposed as the mechanism of pathogenesis and a 19-20 nucleotide sequence similarity between the pathogenicity region of PSTVd and a putative transcription factor (GenBank BI969092) suggested that PSTVd may silence regulatory genes [22]. Enhanced accumulation of viroid-specific siRNAs [23-27], and a bifunctional nuclease and RNase that may be involved in the regulation of plant development [25] were found to be associated with viroid infection in PSTVd-infected tomato plants.

Phosphorylation/dephosphorylation of host proteins most likely also plays a role in viroid pathogenicity. CEVd infection of tomato plants was found to alter the general pattern of leaf protein phosphorylation [28]. PSTVd infection of tomato induced the phosphorylation of a host-encoded 68,000 Mr protein immunologically related to the mammalian ds-RNA dependent protein kinase (PKR) [29-31]; however, the plant ortholog of PKR has not been isolated. PSTVd strains varying in pathogenicity, on the other hand, resulted in differential activation of the mammalian PKR *in vitro*, suggesting a possible triggering event in viroid pathogenesis [32].

**Figure 1:**
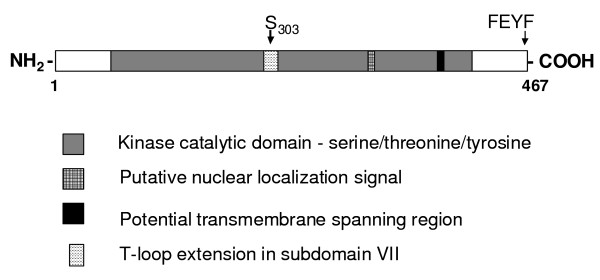
**Schematic diagram showing the key features of PKV**. The protein kinase catalytic domain, putative nuclear localization signal and transmembrane domains, and T-loop extension (in subdomain VII) are indicated by boxes. S_303 _= location of putative phosphorylated serine in the extension loop; FEYF, location of the COOH-terminal FEYF PIF motif. (http://predictprotein.org, [64])

We previously identified a protein kinase gene that was transcriptionally-activated in leaves of tomato plants infected with intermediate and severe strains of PSTVd, but whose transcripts were barely detectable in mock-inoculated plants [33]. The gene, *pkv *(protein kinase viroid-induced; GenBank Accession no. AF143505) encodes a dual-specificity 52 kDa serine-threonine protein kinase (PKV). In addition to protein kinase signature motifs, PKV contains a putative nuclear localization signal and a potential transmembrane spanning region (indicated in Figure 1), seven potential internal myristoylation sites, one N-glycosylation site and one glycosaminoglycan attachment site (not shown). Although PKV has been shown to possess phosphorylation activity *in vitro *[33], it is not known if the other predicted sequence motifs are functional.

PKV is a novel member of the AGC VIIIa protein kinase superfamily [33], members of which have significant homologies to cyclic nucleotide-dependent protein kinases, however, little is known about the role of AGC VIIIa kinases in plants. Within subgroup AGC VIIIa, only PINOID [34] and Adi3 [35] have been genetically characterized, and have been shown to play fundamental roles in auxin signalling and plant cell death, respectively.

To better understand the biological role of PKV in plant development, we introduced sense and antisense copies of the full-length *pkv *gene into experimental *Nicotiana tabacum *cv. Xanthi tobacco plants by stable transformation. *N. tabacum *encodes a homolog of PKV, with 99% sequence identity to the tomato gene (GenBank EU196240), which is also transcriptionally activated to a low level in PSTVd-infected tobacco plants (tobacco is not a symptomatic host of PSTVd; data not shown). We demonstrate that over expression of PKV in tobacco results in dwarfing and reduced root formation, similar to symptoms of PSTVd infection of tomato plants. Results from hormone supplement and gene expression studies suggest that gibberellic acid biosynthetic and/or signalling pathways are regulated by PKV.

## Results

### Molecular analysis of transgenic plants

To determine the role of *pkv *gene expression in plant development, a full-length copy of the open reading frame [33] was cloned in the sense and antisense orientations into the binary vector, pBI121, where transcription of the inserted gene is under control of the CaMV35S promoter. Several independently transformed lines for each construct were obtained with similar phenotypes, and three lines of each construct was selected for further investigations. Southern blot analysis revealed that plants contained a single insert and segregated with a 3:1 ratio (data not shown).

**Figure 2:**
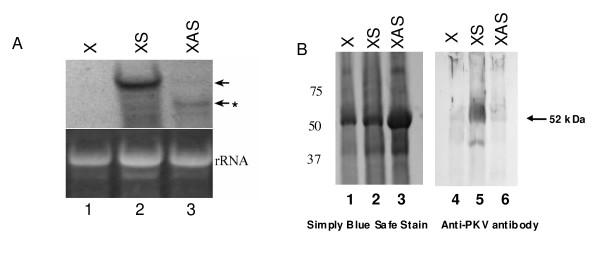
**Characterization of transcript and protein expression in transgenic plants**. **(A) **Northern blot analysis of the accumulation of *pkv *transcripts in transgenic plants as described in the Methods section. Left panel, 20 ug of total RNA extracted from control (1, X), XS (2) and XAS (3). Ethidium bromide staining of ribosomal RNA (rRNA) is shown to illustrate equal loading of RNAs. Arrows indicate location of mRNAs hybridizing to the PKV DIG-labeled probe. Asterisk indicates degraded PKV transcript in XAS plants. (**B) **Western blot analysis of PKV protein accumulation in mature leaves of control and transgenic plants using a PKV-specific polyclonal antibody as described in the Methods section. Equal amounts of total protein were loaded per sample lane. Numbers to the left of the figure represent the size of prestained protein markers. Lane 1, X; Lane 2, XS; Lane 3, XAS. The arrow designates the location of the 52 kDa PKV protein.

Transcript and protein levels of PKV in control and transgenic lines were examined by northern and western blot analyses, respectively (Figure 2). *pkv *transcripts were readily apparent in sense (XS) plants as opposed to untransformed (X) plants (lane 2 and 1, respectively, Figure 2A). By contrast, there was evidence of reduced accumulation and corresponding degradation of the introduced *pkv *transcripts in the antisense (XAS) plants (lane 3, Figure 2A), an indication of gene silencing of the tobacco homolog of PKV and resulting from formation of double-stranded RNAs of target gene transcripts and the introduced antisense gene transcripts. Western blot analysis of PKV protein synthesis revealed the presence of a protein band of approximately 52 kDa reacting to PKV-specific antiserum in XS plants (Figure 2B, lane 5). Although tobacco contains a PKV homolog, no detectable PKV-related protein was evident in the non transgenic control (X) or XAS plants, indicating very low levels of basal expression, similar to what was found in healthy tomato plants [33].

### Phenotypic changes in tobacco in response to transgenic expression of pkv

**Figure 3:**
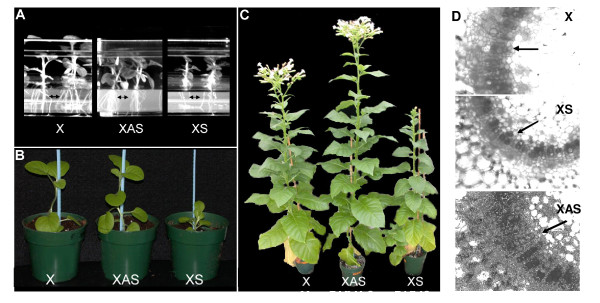
**Growth of transgenic tobacco plants expressing the sense (XS) or antisense (XAS) copy of *pkv***. (**A**) Three week-old *in vitro *plantlets of control (X), XS and XAS showing the reduced (XS) or enhanced (XAS) root production (arrows). (**B**) Five-week old plants in soil showing the dwarfing phenotype of XS. (**C**) Fully mature greenhouse-grown plants showing the increased height and flowering of XAS and the dwarfing habit and reduced/delayed flowering of XS. (**D**) Transverse petiole sections from leaves of mature control (X), XS, and XAS tobacco plants. Sections were observed by light microscopy without staining. Petioles of the fifth leaf from the base were taken from each plant. Original magnification, 100 ×. Arrows designate the columns of lignified xylem elements.

Tobacco plants transformed with the XS and antisense XAS constructs exhibited dramatic alterations in appearance and development compared to non-transformed control plants (X) (Figure 3). XS plants were dwarfed and had noticeably reduced root systems in tissue culture and as young plants (Figure 3A and 3B), phenotypes that are similar to symptoms observed in tomato plants infected by PSTVd. XAS transformants, on the other hand, were taller than non-transformed tobacco plants and had a more extensive root system (not shown in potted plants). The phenotypic responses of the transformants were most pronounced in mature tobacco plants, where XS plants were stunted, possessed thick, dark leaves, and exhibited reduced flowering and root formation, and XAS plants were taller and had increased numbers of inflorescences and flowers (Figure 3C). Morphometric analysis of the mature plants (Table 1) revealed that internode number and stem length were decreased in XS plants and increased in XAS plants. The internode length contributed to the overall difference in height from the non-transformed control. Visualization of the vascular tissue in cross-sections of leaf petioles of control and transgenic plants revealed increased numbers of lignified xylem elements (darker cells) in XAS petioles as compared to petioles derived from similarly positioned leaves of X plants, whereas there was a decreased number of lignified elements in XS plants (Figure 3D).

**Table 1:** Morphometric analysis of fully-grown untransformed (X), PKVS and PKVAS plants.

	X	XAS	XS
Plant size (mm)	989.3 ± 16.67	1219.33 ± 9.94^a^	700.67 ± 52.51^a^

Internode #	71.67 ± 1.76	91.33 ± 0.8^a^	44.6 ± 0.8^a^

Size of 10th internode (cm)	3.9 ± 0.15	5.0 ± 0.03^a^	1.86 ± 0.1^a^

# flowers	42 ± 1.45	65.6 ± 3.8^a^	17.6 ± 1.8^a^

Flower size (cm)	4.46 ± 0.03	4.43 ± .06	3.67 ± .16^a^

Stigma (cm)	3.5 ± 0.1	3.5 ± 0.2	1.63 ± .13^a^

Style (cm)	3.72 ± .02	3.75 ± .1	2.2 ± .1^a^

Pollen (T_1_)	viable	viable	not viable

^a^Values are significantly different compared to the X control (*P *< 0.05) as measured using the

unpaired *t *test. Six plants of each line were used for the analysis with three lines per construct.

Total height and number of internodes was measured from the base of the plant to the base of the inflorescence when all of the flowers were developed.

**Figure 4:**
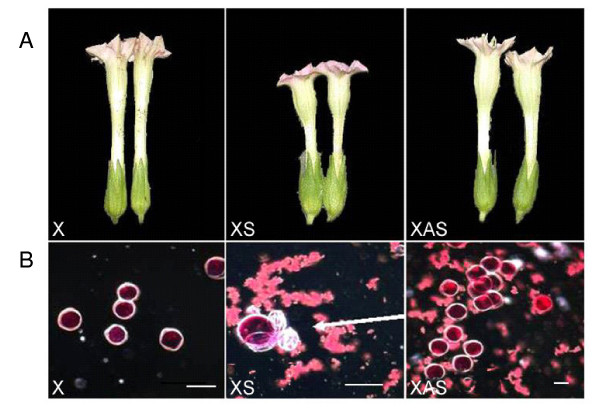
**Morphology of flowers and pollen from of transformants**. (**A**) Over expression of *pkv *results in reduced flower size (XS) as compared to control (X) and XAS. (**B**) Light microscopy of acetocarmine-stained pollen from control (X), XS, and XAS anthers. The arrow in XS points to defective pollen grains which appear white and collapsed in the picture and are adjacent to a viable pollen grain which appears red in the picture. Bar = 20 μm.

Flower size was smaller in XS plants (Figure 4A and Table 1) with resulting shorter style and stigma. In addition, XS plants had greatly reduced fertility, while seed set in XAS plants was not affected. When pollen viability was examined by staining with acetocarmine, the cytoplasm of non-transgenic and XAS pollen stained red, indicating viability, while the majority of the XS pollen of T2, homozygous, selfed plants had collapsed and was nonviable (arrows, Figure 4B). When the XS flowers were pollinated with X pollen, however, the plants produced more seeds than the XS parent, indicating that the XS plants were male sterile but female fertile. Fifty per cent of the seeds resulting from the X × XS cross germinated.

### Northern blot analysis of salicylic acid- and jasmonic acid-signaling pathways in transgenic plants

**Figure 5:**
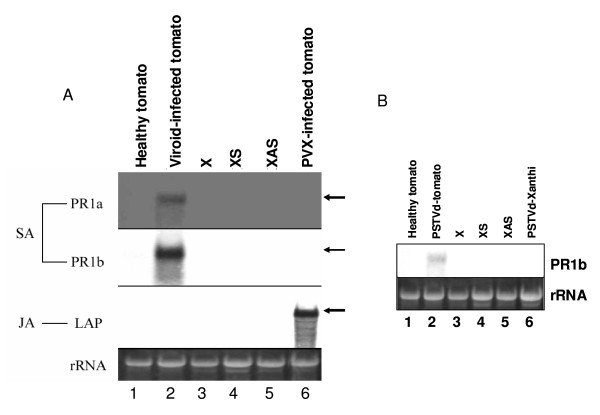
**Northern blot analysis of the expression pattern of salicylic acid- and jasmonic acid- regulated genes**. **(A) **Northern blot analysis of the accumulation *Pr1a, Pr1b*, and LAP transcripts in 20 μg of total RNA extracted from healthy tomato (lane 1), PSTVd-infected tomato (Viroid-infected tomato, lane 2), non-transformed control (X, lane 3), transgenic plants XS (lane 4) and XAS (lane 5), and tomatoes infected with *Potato virus *X (PVX-infected tomato, lane 7). Ethidium bromide staining of ribosomal RNA (rRNA) is shown to illustrate equal loading of RNAs. SA, salicylic acid-inducible genes; JA-jasmonic acid-inducible gene. (**B**) Northern blot analysis of total RNA samples as in (**A**) using only the Pr1b probe. Lane 1, Healthy tomato; Lane 2, PSTVd-infected tomato; Lane 3, X; Lane 4, XS; Lane 5, XAS; Lane 6, PSTVd-infected Xanthi tobacco.

Induced defense and/or pathogenesis-related (PR) gene expression occurs in PSTVd-infected, dwarfed tomato plants [15]. To determine if PR expression is associated with stunting caused by over expression of PKV in tobacco, we probed Northern blots containing total RNA isolated from leaf tissue for transcripts of *Pr1a1 *and *Pr1b*, whose expression is salicylic acid-mediated. These genes were transcriptionally activated in viroid-infected tomato plants, as previously reported, but not in XS or XAS transformants, nor in PSTVd-infected tobacco plants, indicating that neither PSTVd infection nor PKV over expression induce a general pathogenesis response in tobacco (compare lane 2 with lanes 4 and 5 in Figure 5A and lane 2 and lanes 4-6 in Figure 5B). In addition, transcripts of the leucine aminopeptidase gene (*LAP*) (jasmonic acid-mediated; [36]) did not increase in transgenic tobacco plants, nor in viroid-infected tomato (whereas such transcripts did accumulate in *Potato virus *X-infected tomato), indicating that neither the dwarfing caused by PKV over expression in tobacco nor PSTVd pathogenesis in tomato involve jasmonic acid signaling (Figure 5A).

### Exogenous application of gibberellic acid reverses stunting in plants over expressing PKV

**Figure 6:**
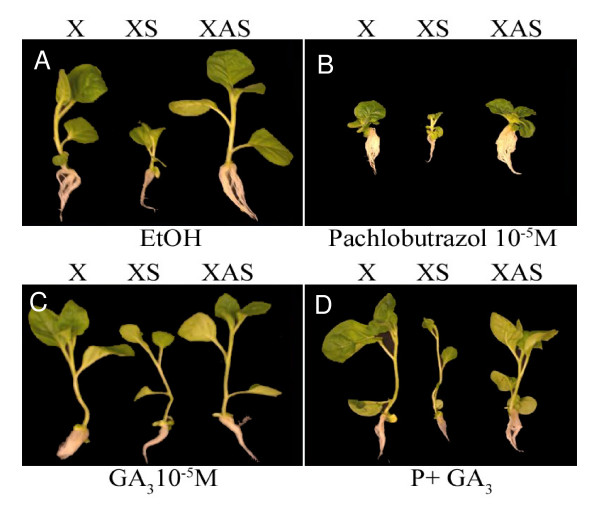
**Growth effects of *in vitro *application of hormone to control and transgenic seedlings**. Ethanol (EtOH), GA_3 _(10^-5^M), pachlobutrazol (10^-5^M), or GA_3_(10^-5^M) plus pachlobutrazol (P) (10^-5^M) were added to solidified media. Plants were evaluated at 20 days after seeding.

To investigate whether the reduced stature of XS plants was due to a lower content of GA or to reduced response to GA, exogenous hormone applications were made to seedlings grown *in vitro *and seedling growth was monitored. The hypocotyl length of XS seedlings increased significantly in medium containing 10^-5 ^M GA_3_, where it was not as greatly affected in control (X) or XAS seedlings (Figure 6, panel C). In medium containing 10^-5 ^pachlobutrazol, an inhibitor of GA biosynthesis, the height of both control and transgenic seedlings was reduced, and this inhibition was fully reversed by addition of 10^-5 ^M GA_3 _(Figure 6, panel D). Therefore the plants respond normally to GA. This suggests that the dwarf phenotype of XS plants is due to a lower content of active GAs. The phenotype of mature XAS plants suggests that these plants may be producing more GA than the control, non-transformed plants

### Transcript levels of genes involved in GA biosynthesis and leaf expansion

**Figure 7:**
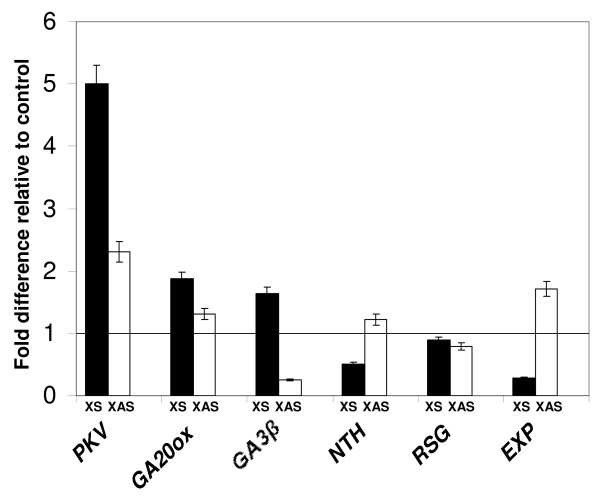
**Real-time quantitative RT-PCR (QPCR) analysis of the effects of transgenes on the expression of host genes**. Total RNA isolated from leaf tissue of X, XS, and XAS plants was subjected to QPCR analysis using primers specific for the indicated genes as described in the Methods section. Relative transcript expression levels of each target were normalized with respect to actin and the transgenic values reflect fold change expression compared to non-transgenic (X) controls. Six biological replications were used to calculate mean values and standard deviations. Bars mark the standard deviation of the average. The line across the figure indicates the normalized level of 1 in the non-transgenic control.

The dwarfing phenotype of XS plants and its reversal by exogenous GA_3 _treatment suggested defects in GA biosynthesis [37]. The accumulation of mRNAs of genes involved in GA biosynthesis, GA20ox1 (GA20ox) and GA3β-hydroxylase (GA3β), in non-transformed (X), XS, and XAS tobacco plants was evaluated by real-time quantitative RT-PCR (Figure 7). GA20ox and GA3β transcript levels were 1.6-1.8 fold higher in XS plants compared to X or XAS. In XAS plants, GA3β transcript levels were reduced significantly compared to the non-transformed control. It has been reported that, as key enzymes in the last catalytic steps in the synthesis of active GA's, GA3β and GA20ox are subject to feedback regulation by GA [37]. Therefore, in XS plants, low levels of GA could result in induction of these enzymes, whereas their gene expression would be repressed in plants containing higher levels of GA.

Endogenous GA levels have also reported to be modulated by the tobacco knotted-1 homeobox gene *NTH *(*Nicotiana tabacum *homeobox gene 15) [38] and the tobacco bZIP transcriptional activator gene *RSG *(repression of shoot growth) [39], which regulates the transcript levels of GA biosynthetic enzymes. Over expression of the *NTH15 *gene in tobacco has been found to lead to decreased transcript levels of GA20ox and to abnormal leaf morphology with little or no effect on GA3β transcript levels [38]. In our study, *NTH *transcript levels were down-regulated in XS plants compared to non-transformed control, and were up-regulated in XAS plants (Figure 7). The dominant-negative form of *RSG *represses the expression of the *ent*-kaurene oxidase gene in the GA biosynthetic pathway, reducing GA levels, and resulting in a dwarfing phenotype in tobacco [39]. In our studies, *RSG *levels in X, XS, and XAS plants appeared to be similar to one another (Figure 7).

The change in plant height in XS or XAS plants could be due to changes in cell number or cell size. Transcript levels of *LeExp2 *(EXP), the major expansin gene expressed in stems and hypocotyls of tomato and regulated by auxin, have been shown to correlate with growth rate in rapidly expanding hypocotyl tissue due to increased cell size [40]. In our analysis of EXP transcript levels in tobacco leaf tissue using real time RT-PCR (experiment was repeated several times), there was a positive correlation between plant and leaf size and EXP expression in XS and XAS plants, with EXP transcripts expressed at 0.28 fold in XS plants as compared to non-transgenic plants (X), while EXP transcripts were up-regulated and in XAS plants with respect to non-transgenic plants (Figure 7). This result confirms what was reported by Qi and Ding [17] in dwarfed, viroid-infected plants.

## Discussion

### Constitutive sense and antisense expression of PKV affects plant development

Our results suggest that PKV regulates plant development by modulating levels of GA and the enzymes involved in GA biosynthesis. Constitutive over expression of PKV resulted in visually obvious phenotypes of stunting and reduced root formation. In addition, the plants were male infertile which may be attributed to defective pollen. Accumulation of PKV transcripts or protein in flowers may disrupt pollen production by affecting development of stamens and microspore mother cells, resulting in nonviable pollen. Antisense expression of *pkv *(XAS), on the other hand, resulted in plants that were taller than non-transformed plants, with enhanced root development and greater numbers of flowers in the inflorescence, but with no obvious influence on seed set.

The abnormal development induced by constitutive over expression of PKV resembles that found in viroid-infected plants. For example, reduced flower size was evident in *Hop stunt viroid *(HSVd)-infected cucumber [41] and in chrysanthemum infected with *Chrysanthemum stunt viroid *[42]. In addition to stunting, infection of tomato by severe strains of PSTVd reduced pollen viability [41]. Symptoms of viroid infection in tissue culture are varied but include specific inhibition of root formation by HSVd in cucumber [43] and root and shoot formation by CEVd in tomato [44]. Finally, vascular system development was arrested in CEVd-infected tomato plants [45].

### Changes in pkv expression result in altered expression levels of GA biosynthetic genes

Many factors are responsible for dwarfing phenotypes of plants, but the roles of GA, auxins, and brassinosteroid hormones are the most studied [46]. These hormones control separate processes that contribute to stem elongation, and a deficiency due to reduced synthesis of the hormones or the lack of response to the hormones may lead to a dwarf phenotype. The elongation response of XS seedlings to exogenous GA suggests that they contain lower levels of active GA and are not impaired in their response to the hormone (Figure 4). Altered GA levels impact lignin biosynthesis and development of xylem vessels in tobacco plants [47]. The reduced number of xylem vessels in the petioles of XS plants and increased number in XAS plants also suggests decreased and increased levels of active GA's, respectively (Figure 2D).

GA20ox1 and GA3β hydroxylase are key biosynthetic enzymes in the pathways to active GA_4 _and GA_1 _and are subject to feedback regulation [48]. The transcripts of these genes are up-regulated in GA-deficient XS plants, as might be expected (Figure 7). Positive and negative signaling components in GA signal transduction have been characterized, and include the DELLA proteins and bZIP transcriptional activators and repressors [37,49]. Further investigations of transcriptional activation of enzymes and activators/repressors involved in GA biosynthesis and catabolism will help unravel the biochemical pathways responsible for the dwarfing phenotype in XS plants.

Because of the reduced height of XS plants and increased height of XAS plants, we would expect lower and higher levels, respectively, of *LeExp2 *(EXP) transcripts in these plants. As shown in Figure 7, EXP transcript levels as evaluated are reduced in leaves of XS plants compared to control and XAS plants. As the *LeExp2 *gene is expressed primarily in stems and hypocotyls of plants [40], the reduced leaf size in XS and enlarged leaf size in XAS also result from altered *LeExp2 *gene expression.

*NTH15*, a knotted-1 homeobox gene, and the bZIP transcriptional activator gene *RSG *modulate endogenous GA levels by regulating the expression of GA biosynthetic enzymes. For example, over expression of *NTH15 *decreases the expression of GA20ox1, leading to abnormal leaf and flower morphology. Following that reasoning, the results in Figure 7 showing that down regulation of *NTH *expression levels corresponds to an increase in GA20ox1 transcripts in XS plants. These results suggest the NTH may be involved in PKV signaling.

The RSG protein binds and activates the promoter of *GA3*, encoding *ent*-kaurene oxidase, another key enzyme in the GA biosynthetic pathway. Expression of a dominant-negative form of *RSG *gene in tobacco plants severely inhibits stem internode growth [39]. In our study, *RSG *transcript levels are not repressed in XS plants, suggesting that RSG may not play a role in PKV signaling However, as RSG is activated through phosphorylation [39], the lack of regulation at the transcriptional level does not eliminate its involvement in the regulation of endogenous GA levels in XS and XAS plants. Further investigations of transcriptional activation of enzymes and activators/repressors involved in GA biosynthesis and catabolism will help unravel the biochemical pathways responsible for the dwarfing phenotype in XS plants.

### Proposed functional role of PKV in plant development and viroid pathology

PKV is a member of the AGC protein kinases (specifically Group AGC VIIIa), members of which have been shown to play roles in growth signaling pathways, regulation of transcription, and programmed cell death [50]. Key features of the AGCVIIIa family, and shared by PKV and related kinases, include the DFD motif in the activation loop, a 50-80 amino acid variable insertion (T-loop extension) in subdomain VII of the conserved catalytic domain, and a C-terminal hydrophobic domain known as PIF (PDK1 [3-phosphoinositide-dependent protein kinase 1]-interacting fragment), characterized by the amino acid motif FxxFs/TF/Y (represented by FEYF in PKV) (Figure 1). Two members of the AGC VIIIa family, *PINOID*, which regulates organ development by enhancing polar auxin transport in plants [34,51], and Adi3, which negatively regulates plant cell death [35] are activated by PDK1 phosphorylation [52-55]. PKV activity may also be regulated by PDK1 phosphorylation. PDK1 interaction with PKV through the PIF domain at the carboxy terminus of PKV (Figure 1) would result in phosphorylation of the serine at position 303 in the T-loop extension of PKV (Figure 1), thereby transactivating PKV and leading to autophosphorylation at additional sites. Phosphorylated and activated PKV would subsequently transphosphorylate downstream transcriptional activators or repressors, or genes encoding enzymes involved in GA biosynthesis or degradation, resulting in stunted plants exhibiting morphogenic abnormalities that characterize viroid-induced disease.

Unlike viroid- or virus-infected tomato plants, transcripts of pathogenesis-related proteins were not induced in *pkv *sense-transformed tobacco plants, suggesting that *pkv *downstream signaling is not through salicylic acid- or jasmonic acid-dependent pathways. The putative promoter region of *pkv *contains G- (CACGTG) and H-box (GGTAGG) *cis *elements [33] which are known to interact with, and be transcriptionally activated by, bZIP transcriptional factors. These *cis *elements are speculated to be responsible for early responses to pathogen attack [56], but they also function in the regulation of genes by developmental stimuli [57]. The genes encoding PKV and the PR proteins may be transcriptionally activated by the same bZIP transcription factors but diverge into separate signaling pathways in tomato. Further studies are needed to determine the extent to which PSTVd infection regulates the transcription of *pkv *and to dissect the signaling pathways and downstream substrates of PKV phosphorylation. PSTVd RNA may bind to the *pkv *promoter directly and enhance transcription. Alternatively, because of its partially double-stranded structure [1], viroid RNA could trigger posttranscriptional gene silencing of plant transcriptional activators or repressors, as proposed by Wang et al. [22], or viroid RNAs could act as transcriptional activators by targeting the noncoding regulatory regions in gene promoters. Alternatively, PSTVd RNA may bind to the *pkv *promoter directly and enhance transcription. Although the exact mechanism of transcriptional activation by short dsRNAs is unknown, Li et al. [58] demonstrated this mechanism in human cells. Further studies are needed to determine the extent to which PSTVd infection regulates the transcription of *pkv *and to dissect the upstream signaling components, including PDK1, and and downstream substrates of PKV phosphorylation to dissect the role of PKV in plant development.

## Conclusion

In this study, we have found that the over expression of the tomato serine-threonine protein kinase gene PKV resulted in stunting, modified vascular tissue development, reduced root formation, and male sterility in tobacco, while silencing of the endogenous tobacco PKV gene by antisense expression resulted in a taller stature and increased numbers of influorescences in transgenic plants. The combined results of reversal of stunting in plants over expressing PKV by exogenous application of GA and the altered transcript levels of GA biosynthetic genes suggests that PKV regulates plant development by functioning in critical signaling pathways involved in GA metabolism and GA-regulated transcriptional networks. Our findings establish a foundation to further investigate the molecular mechanisms of how PKV interacts with critical cellular factors, including transcriptional regulators such as DELLA-domain proteins [59], that lead to altered plant development. Ultimately, it may also lead to a greater understanding of the signaling pathways functioning in viroid pathogenicity.

## Methods

### DNA manipulations

PKV sense (GenBank accession no. AY849915; 33) and PKV antisense sequences were cloned into the binary vector pBI121 (Clontech, Inc., Palo Alto, CA) at the *Bam*HI/*Sst*I sites, releasing the GUS gene. For preparation of the sense insert, construct pGEXKG/PKV [33] was doubly digested with *Bam*HI and *Sst*I, and the PKV sense fragments were recovered. To prepare the antisense insert, construct pET28a/PKV [33] was first digested with *Eco*RI to release the PKV insert, which was then ligated back into the pET28a vector at the *Eco*RI site, generating clones with opposite orientations. Antisense clones were selected and one of the clones was doubly digested with *Bam*HI and *Sst*I, giving rise the antisense insert, which contained cohesive ends compatible to the above prepared pBI121 vector. The pBI121 constructs were transformed into *Agrobacterium tumefaciens *LBA4404.

### Plant growth conditions

Tobacco plants were grown under greenhouse conditions under natural daylight with a temperature of 20-25°C.

### Plant transformation and hormone treatments

Leaf pieces of *Nicotiana tabacum *cv. Xanthi were transformed with a suspension of *A. tumefaciens *LBA4404 bacterial cells containing the pBI121 PKV constructs as described by Horsch et al. [60]. Stable incorporation of the genes was verified by either Southern blot hybridization or PCR analysis. Plant height, internode length, number of flowers, and flower size were measured from three plants of each category and the data were compared using the unpaired *t *test. Seeds of control and transgenic tobacco plants were germinated and grown in Petri dishes containing solidified sterile 1/2 MS media containing 3% sucrose and either 10^-5 ^M GA_3_, 5 μM indolebutyric acid (Sigma Chemical Co., St Louis, MO) or 10^-5 ^M pachlorbutrazol (*Phyto*Technology Laboratories, Shawnee Mission, KS), alone or in combination, or ethanol as a control was added at the same concentration as used to solubilize the hormones.

### Northern, Southern, and western blot analyses

Total RNAs were extracted from plant tissue using Tri Reagent (Molecular Research, Cincinnati, OH). Twenty μg aliquots were treated with glyoxyal using the Northern max RNA kit (Ambion, Houston, TX) and were electrophoresed through 1% agarose gels and blotted onto nylon membranes following manufacturers' instructions. DNA clones corresponding to the *Pr1a1 *and *Pr1b *genes were prepared by RT-PCR amplified from tomato total RNA using Titan One-tube RT-PCR (Roche Molecular Biochemicals, Bedford, MA) and primers PKVexonF and PKVexonR corresponding to the tobacco PKV gene *pkv; *primers Pr1aF and PrR, corresponding to the *Pr1a1 *gene [11]; and primers Pr1b and PrR, corresponding to the *Pr1b *gene [61] (primers sequences listed in Table 2). The PCR products were cloned in the pCR2.1 TA cloning vector (Invitrogen, Carlsbad, CA). PCR products were sequenced to confirm their identity. DIG-labeled DNA probes were produced from these cDNA clones using the primers listed above and the DIG High-Prime DNA labeling kit (Roche Molecular Biochemicals) following manufacturer's instructions. Primers LAPF and LAPR were used to prepared a 397 bp DIG-labeled DNA probe from a pUC119:leucine aminopeptidase cDNA clone [[62]; gift of L. Walling].

**Table 2:** Oligonucleotide primers used in experimental analyses.

Oligonucleotide	Sequence	**Accession No**.	Gene	Amplicon, bp
PKVexonF	5'-GAAATCCTAGCAGTGGATCGG-3'	EU196240	*pkv*	531

PKVexonR	5'-CAGCACTTCTTACTAAAGCCC-3'	"	"	"

Pr1a	5'-CAAACTCCTCGAGAGAATTT-3'	X71592	*Pr1a1*	299

PrR	5'-ACCACTTGAGTATAATGTC-3'	"	"	"

Pr1bF	5'-CAAAATTCACCCCAAGACTA-3'	X12486	*Pr1b*	300

LAPF	5'-ATGCAGAACATGTATGTGCAG-3'	SLU50151	*LAP*	397

LAPR	5'-TTTGCTGCACCTAAAACAGC-3'	"	"	"

ACTRQF	5'-GTGGCGGTTCGACTATGTTT-3'	EU938079	*ACTIN1*	186

ACTRQR	5'-ATTCTGCCTTTGCAATCCAC-3'	"	"	"

PKVRQF	5'-TCCGTTGTTCTGTCAATCCA-3'	EU196240	*pkv*	208

PKVRQR	5'-CCTCCCACAAAAAGACCAAA-3'	"	"	"

GA20RQF	5'-TTCCGGTTCCACTTATCGAC-3'	AB032198	*GA20 oxidase 1*	153

GA20RQR	5'-GGCGTTGGAGATGATATTA-3'	"	"	"

GA3RQF	5'-TCAAAGAAGGGAGTGGTTGG-3'	AB032198	*GA3β hydroxylase*	155

GA3RQR	5'-GGCTACAGAAAGGCGATGTC-3'	"	"	"

NTH15RQF	5'-CCCTCAGGCTGAAGATCAAG-3'	AB004785	*NTH15*	152

NTH15RQR	5'-GTCTGGTCCACCAGTCCAGT-3'	"	"	"

RSGRQF	5'-TGCTGAGTTGGCTTTGATTG-3'	AB040471	*RSG*	242

RSGRQR	5'-CTCCTTGTTCCAAAGCTTGC-3'	"	"	"

EXPRQF	5'-TGTTGGAGGTGCTGGTGATA-3'	AF096776	*LeExp2*	215

EXPRQR	5'-CCCCTCAAAAGTTTGTCCA-3'	"	"	"

Northern blot hybridizations were carried out in a DIG Easy Hybridization buffer (Roche Molecular Biochemicals) at 47°C overnight. The blots were washed for 2 × 15 minutes in 2 × SSC (1 × SSC is 0.15 M NaCl, 0.015 M sodium citrate, pH 7.0), 0.1% SDS at room temperature, followed by 2 × 15 minutes washed in 0.5 SSC, 0.1% SDS at 68°C. Colorimetric detection was made using the DIG wash and block buffer set (Roche Molecular Biochemicals) and NBT/BCIP according to manufacturer's instructions (Kirkegaard and Perry, Gaithersburg, MD). For Southern blot analysis, RT-PCR products were electrophoresed through a 1% agarose/TBE gel, blotted onto a nitrocellulose membrane, and hybridized using the same conditions used for Northern blot analyses [33].

Total proteins were extracted from plant tissues using a 2:1 ratio (buffer: tissue) of Cell- Lytic P Plant Cell Lysis/Extraction Reagent (Sigma Chemical Company) containing a 1:100 dilution of Plant Protease Inhibitor cocktail (Sigma Chemical Company) by grinding in an eppendorf tube using a disposable pestle. Samples were centrifuged for ten minutes at 16,000 × g in a microfuge. Laemmli sample buffer (Bio-Rad Laboratories, Hercules, CA) was added (2:1, sample/dye ratio), the sample was boiled for 10 minutes, and a 20 μl aliquot was electrophoresed under denaturing conditions through a 10-20% Tris-glycine gel (Invitrogen) and blotted according to manufacturers instructions onto a nitrocellulose membrane (Invitrogen). The size marker was the Precision Plus Kaledioscope prestained markers (Bio-Rad Laboratories). Western blot analysis was carried out using a polyclonal antibody raised against a PKV-GST fusion protein [33] (Cocalico Inc, Reamstown, PA) and a secondary goat anti-rabbit polyclonal antibody (Kirkegaard and Perry). Colorimetric detection was performed using NBT/BCIP according to manufacturers' instructions (Kirkegaard and Perry).

### Quantitative real-time RT-PCR analysis (QPCR)

Total RNA was extracted from plant tissues using TriReagent. First strand cDNA was prepared using an oligo(dT) primer from 1 μg total RNA using the Advantage RT-for-PCR kit (Clontech). The cDNA reaction was diluted to 100 μl with sterile water. QPCR was performed using the first-strand cDNA generated above, selected primer sets designed using the Primer3 program [63] and listed in Table 2 as RQ primers, and the Brilliant^® ^SYBR^® ^Green QPCR Master Mix (Stratagene, La Jolla, CA). Each QPCR reaction contained 12.5 μl of 2× Brilliant^® ^SYBR^® ^Green QPCR Master Mix, 2 ul of the diluted cDNA, and 2.5 pM of each gene-specific primer. The conditions used for the QPCR reactions were: 95 C for 10 min, followed by 40 cycles of 95 C, 30 sec, 50 C, for 1 min, and 72 C for 30 sec in a Mx3000P QPCR System (Stratagene). Data were analyzed using the MxPro software (Stratagene) and relative transcript expression levels of each target were normalized with respect to the tobacco *ACTIN *gene (Accession no. EU938079). Six biological replications were used to calculate mean values and standard deviations.

### Microscopy

Pollen grains were stained with acetocarmine and examined under light microscopy in a Zeiss Axioskop 2 microscope (Carl Zeiss, Inc., Thornwood, NY). Hand-cut sections of leaf petioles were examined using light microscopy.

## Authors' contributions

RWH and YZ carried out the cloning of recombinant DNA constructs, genetic transformation of plants, and phenotypic and molecular characterization of the transgenic plants. RWH and YZ designed the strategy, analysis of data and interpretation, and drafted the manuscript.

## References

[B1] Diener TO Ed The Viroids 1987 344 Plenum Press, New York

[B2] Owens RA Blackburn M Ding B Possible involvement of the phloem lectin in long-distance viroid movement Mol Plant Microbe Interact 2001 14 905 909 10.1094/MPMI.2001.14.7.905 11437264

[B3] Harders J Lukacs N Robert-Nicoud M Jovin TM Riesner D Imaging of viroids in nuclei from tomato leaf tissue by in situ hybridization and confocal laser scanning microscopy EMBO J 1989 8 3941 3949 2591366 10.1002/j.1460-2075.1989.tb08577.x PMC401569

[B4] Qi Y Ding B Differential subnuclear localization of RNA strands of opposite polarity derived from an autonomously replicating viroid Plant Cell 2003 15 2566 2577 10.1105/tpc.016576 14555700 PMC280561

[B5] Schnölzer M Haas B Ramm K Hofmann H Sänger HL Correlation between structure and pathogenicity of potato spindle tuber viroid (PSTVd) EMBO J 1985 4 2181 2190 15938051 10.1002/j.1460-2075.1985.tb03913.x PMC554484

[B6] Rodriguez JL Garcia-Martinez JL Flores R The relationship between plant growth substances content and infection of *Gynura aurantiaca *DC by citrus exocortis viroid Physiol Plant Pathol 1978 13 355 363 10.1016/0048-4059(78)90052-8

[B7] Vera P Hernandez-Yago J Conejero V "Pathogenesis-related" P1 (p14) protein. Vacuolar and apoplastic localization in leaf tissue from tomato plants infected with citrus exocortis viroid: In vitro synthesis and processing J Gen Virol 1989 70 1933 1942 10.1099/0022-1317-70-8-1933

[B8] Vera P Tornero P Conejero V Cloning and expression of a viroid-induced peroxidase from tomato plants Mol Plant-Microbe Interact 1993 6 790 794 8118059

[B9] Ruiz-Medrano R Jimenez-Moraila B Herrera-Estrella L Rivera-Bustamante RF Nucleotide sequence of an osmotin-like cDNA induced in tomato during viroid infection Plant Mol Biol 1992 20 1199 1202 10.1007/BF00028909 1463856

[B10] Rodrigo I Vera P Tornero P Hernandez-Yago J Conejero V cDNA cloning of viroid-induced tomato pathogenesis-related protein P23. Characterization as a vacuolar antifungal factor Plant Phys 1993 102 939 945 10.1104/pp.102.3.939 PMC158867 8278538

[B11] Tornero P Conejero V Vera P A gene encoding a novel isoform of the PR-1 protein family from tomato is induced upon viroid infection Mol Gen Genet 1994 243 47 53 10.1007/BF00283875 8190070

[B12] Tornero P Conejero V Vera P Primary structure and expression of a pathogen-induced protease (PR-P69) in tomato plants: Similarity of functional domains to subtilisin-like endoproteases Proc Natl Acad Sci USA 1996 93 6332 6337 10.1073/pnas.93.13.6332 8692815 PMC39022

[B13] Domingo C Conejero V Vera P Genes encoding acidic and basic class III β-1,3-glucanases are expressed in tomato plants upon viroid infection Plant Mol Biol 1994 24 725 732 10.1007/BF00029854 8193297

[B14] Gadea J Mayda ME Conejero V Vera P Characterization of defense-related genes ectopically expressed in viroid-infected tomato plants Mol Plant-Microbe Interact 1996 9 409 415 8672818 10.1094/mpmi-9-0409

[B15] Itaya A Matsuda Y Gonzales RA Nelson RS Ding B *Potato spindle tuber viroid *strains of different pathogenicity induces and suppresses expression of common and unique genes in infected tomato Mol Plant Microbe Interact 2002 10 990 999 10.1094/MPMI.2002.15.10.990 12437296

[B16] Vidal AM Ben-Cheikh W Talón M García-Martínez JL Regulation of gibberellin 20-oxidase gene expression and gibberellin content in citrus by temperature and citrus exocortis viroid Planta 2003 216 442 448 10.1007/s00425-003-0999-2 14520571

[B17] Qi Y Ding B Inhibition of cell growth and shoot development by a specific nucleotide sequence in a noncoding viroid RNA Plant Cell 2003 15 1360 1374 10.1105/tpc.011585 12782729 PMC156372

[B18] Owens RA Chen W Hu Y Hsu Y-H Suppression of potato spindle tuber viroid replication and symptom expression by mutations which stabilize the pathogenicity domain Virology 1995 208 554 564 10.1006/viro.1995.1186 7747427

[B19] Owens RA Steger G Hu Y Fels A Hammond RW Riesner D RNA structural features responsible for potato spindle tuber viroid pathogenicity Virology 1996 222 144 158 10.1006/viro.1996.0405 8806495

[B20] Owens RA Thompson SM Steger G Effects of random mutagenesis upon potato spindle tuber viroid replication and symptom expression Virology 1991 185 18 31 10.1016/0042-6822(91)90749-2 1926773

[B21] Hammond RW Analysis of the virulence modulating region of potato spindle tuber viroid (PSTVd) by site-directed mutagenesis Virology 1992 187 654 662 10.1016/0042-6822(92)90468-5 1546460

[B22] Wang M-B Bian X-Y Wu L-X Smith NA Isenegger D Wu R-M Masuta C Vance VB Watson JM Rezaian A Dennis ES Waterhouse PM On the role of RNA silencing in the pathogenicity and evolution of viroids and viral satellites Proc Natl Acad Sci USA 2004 101 3275 3280 10.1073/pnas.0400104101 14978267 PMC365780

[B23] Itaya A Zhong X Bundschuh R Qi Y Wang Y Takeda R Harris AR Molina C Nelson RC Ding B A structured viroid RNA serves as a substrate for dicer-like cleavage to produce biologically active small RNAs but is resistant to RNA-induced silencing complex-mediated degradation J Virol 2007 81 2980 2994 10.1128/JVI.02339-06 17202210 PMC1865973

[B24] Machida S Yamahata N Watanuki H Owens RA Sano T Successive accumulation of two size classes of viroid-specific small RNAs in potato spindle tuber viroid-infected plants J Gen Virol 2007 88 3452 3457 10.1099/vir.0.83228-0 18024916

[B25] Matoušek J Kozlová P Orctová L Schmitz A Pešina K Bannach O Diermann N Steger G Riesner D Accumulation of viroid-specific small RNAs and increase in nucleolytic activities linked to viroid-caused pathogenesis Biol Chem 2007 388 1 13 10.1515/BC.2007.001 17214544

[B26] Papaefthimiou I Hamilton A Denti M Baulcombe D Tsagris M Tabler M Replicating potato spindle tuber viroid RNA is accompanied by short RNA fragments that are characteristic of post-transcriptional gene silencing Nucleic Acids Res 2001 29 2395 2400 10.1093/nar/29.11.2395 11376158 PMC55696

[B27] Denti MA Boulta A Tsagris M Tabler M Short interfering RNAs specific for potato spindle tuber viroid are found in the cytoplasm but not in the nucleus Plant J 2004 37 762 769 10.1111/j.1365-313X.2004.02001.x 14871315

[B28] Vera P Conejero V Citrus exocortis viroid alters the in vitro pattern of protein phosphorylation of tomato leaf proteins Mol Plant-Microbe Interact 1990 3 28 32

[B29] Crum CJ Hiddinga HJ Roth DA Tobacco mosaic virus infection stimulates the phosphorylation of a plant protein associated with double-stranded RNA-dependent protein kinase activity J Biol Chem 1988 263 13440 13443 3417665

[B30] Hiddinga HJ Crum CJ Roth DA Viroid-induced phosphorylation of a host protein related to a dsRNA-dependent protein kinase Science 1988 241 451 453 10.1126/science.3393910 3393910

[B31] Meurs E Chong K Galabru J Thomas NSB Kerr IM Williams BRG Hovanessian AG Molecular cloning and characterization of the human double-stranded RNA-activated protein kinase induced by interferon Cell 1990 62 379 390 10.1016/0092-8674(90)90374-N 1695551

[B32] Diener TO Hammond RW Black T Katze MG Mechanism of viroid pathogenesis: Differential activation of the interferon-induced, double-stranded RNA-activated, Mr 68000 protein kinase by viroid strains of varying pathogenicity Biochemie 1993 75 533 538 10.1016/0300-9084(93)90058-Z 7505621

[B33] Hammond RW Zhao Y Characterization of a tomato protein kinase gene induced by infection by *Potato spindle tuber viroid* Mol Plant-Microbe Interact 2000 13 903 910 10.1094/MPMI.2000.13.9.903 10975647

[B34] Christensen SK Dagenais N Chory J Weigel D Regulation of auxin response by the protein kinase PINOID Cell 2000 100 469 478 10.1016/S0092-8674(00)80682-0 10693763

[B35] Devarenne TP Ekengren SK Pedley KF Martin GB Adi3 is a Pdk1-interacting AGC kinase that negatively regulates plant cell death EMBO J 2006 25 255 265 10.1038/sj.emboj.7600910 16362044 PMC1356353

[B36] Ruiz-Rivero OJ Prat S A -308 deletion of the tomato *LAP *promoters is able to direct flower-specific and MeJA-induced expression in transgenic plants Plant Mol Biol 1998 36 639 648 10.1023/A:1005980028203 9526496

[B37] Fleet CM Sun T-P A DELLAcate balance: the role of gibberellin in plant morphogenesis Curr Opin Plant Biol 2005 8 77 85 10.1016/j.pbi.2004.11.015 15653404

[B38] Tamaoki M Kusaba S Kano-Murakami Y Matsuoka M Ectopic expression of a tobacco Homeobox Gene, *NTH15*, dramatically alters leaf morphology and hormone levels in transgenic tobacco Plant Cell Physiol 1997 38 917 927 9327591 10.1093/oxfordjournals.pcp.a029252

[B39] Fukazawa J Sakai T Ishida S Yamaguchi I Kamiya Y Takahashi Y Repression of shoot growth, a bZIP transcriptional activator, regulates cell elongation by controlling the level of gibberellins Plant Cell 2000 12 910 915 10.1105/tpc.12.6.901 PMC149092 10852936

[B40] Caderas D Muster M Vogler H Mandel T Rose JKC McQueen-Mason S Kuhlemeier C Limited correlation between expansin gene expression and elongation growth rate Plant Phys 2000 123 1399 1413 10.1104/pp.123.4.1399 PMC59097 10938357

[B41] Van Dorst HLM Peters D Some biological observations on pale fruit, a viroid-incited disease of cucumber Neth J Plant Pathol 1974 80 85 96 10.1007/BF01980613

[B42] Horst RK Langhans RW Smith S Effects of chrysanthemum stunt, chlorotic mottle, aspermy and mosaic on flowering and rooting of chrysanthemums Phytopathology 1977 67 9 14 10.1094/Phyto-67-9

[B43] Hooker WJ Tai W Yang TC Germination reduction in PSTV infected tomato pollen Am Potato J 1978 55 378 10.1007/BF02852012

[B44] Takahaski T Chiba K Ozaki R Sadakata H Andoh Y Yoshikawa N Growth characteristics in cultured cucumber tissues infected with hop stunt viroid J Phytopathol 1992 136 288 296 10.1111/j.1439-0434.1992.tb01311.x

[B45] Duran-Vila N Semancik JS Effects of exogeneous auxins on tomato tissue infected with the citrus exocortis viroid Phytopathology 1982 72 777 781 10.1094/Phyto-72-777

[B46] Bishopp A Mähönin AP Helariutta Y Signs of change: hormone receptors that regulate plant development Development 2006 133 1857 1869 10.1242/dev.02359 16651539

[B47] Biemelt S Tschiersch H Sonnewald U Impact of altered gibberrellin metabolism on biomass accumulation, lignin biosynthesis, and photosynthesis in transgenic tobacco plants Plant Physiol 2004 135 254 265 10.1104/pp.103.036988 15122040 PMC429367

[B48] Schomburg FM Bizzell M Lee DJ Zeevart JAD Amasino RA Overexpression of a novel class of gibberellin 2-oxidases decreases gibberellin levels and creates dwarf plants Plant Cell 2003 15 151 163 10.1105/tpc.005975 12509528 PMC143488

[B49] Sun TP Gubler F Molecular mechanisms of gibberrellin signaling in plants Ann Rev Plant Biol 2004 55 197 223 10.1146/annurev.arplant.55.031903.141753 15377219

[B50] Bögre L Ökrész L Henriques R Anthony RG Growth signalling pathways in Arabidopsis and the AGC protein kinases Trends Plant Sci 2003 8 424 431 10.1016/S1360-1385(03)00188-2 13678909

[B51] Benjamins R Quint A Weijers D Hooykaas P Offringa R The PINOID protein kinase regulates organ development in *Arabidopsis *by enhancing polar transport Development 2001 128 4047 4067 10.1242/dev.128.20.4057 11641228

[B52] Frödin M Antal TL Dümmler BA Jensen CJ Deak M Gammeltoft S Biondi RM A phosphoserine/threonine-binding pocket in AGC kinases and PDK1 mediates activation by hydrophobic motif phosphorylation EMBO J 2002 21 5396 5407 10.1093/emboj/cdf551 12374740 PMC129083

[B53] Zegzouti H Anthony RG Jahchan N Bögre L Christensen SK Phosphorylation and activation of PINOID by the phospholipid signaling kinase 3-phosphoinositide-dependent protein kinase 1 (PDK1) in *Arabidopsis* Proc Natl Acad Sci USA 2006 103 6404 6409 10.1073/pnas.0510283103 16601102 PMC1458890

[B54] Zegzouti H Li W Lorenz TC Xie M Payne CT Smith K Glenny S Payne GS Christensen SK Structural and functional insights into the regulation of *Arabidopsis *AGC VIIIa kinases J Biol Chem 2006 281 35520 35530 10.1074/jbc.M605167200 16973627

[B55] Galvan-Ampudia CS Offringa R Plant evolution: AGC kinase tell the auxin tale Trends Plant Sci 12 541 547 10.1016/j.tplants.2007.10.004 18024140

[B56] Droge-Laser W Kaiser A Lindsay WP Halkier BA Loake GJ Doerner P Dixon RA Lamb C Rapid stimulation of a soybean protein-serine kinase that phosphorylates a novel bZIP DNA-binding protein, G/HBF-1, during the induction of early transcription-dependent defenses EMBO J 1997 16 726 738 10.1093/emboj/16.4.726 9049302 PMC1169674

[B57] Pastori GM Foyer CH Common components, networks, and pathways of cross-tolerance to stress. The central role of "redox" and abscisic acid-mediated controls Plant Phys 2002 129 460 468 10.1104/pp.011021 PMC1540233 12068093

[B58] Li L-C Okimo ST Zhao H Pookot D Place RF Urakami S Enokida H Dahiya R Small dsRNAs induce transcriptional activation in human cells Proc Natl Acad Sci USA 2006 103 17337 17342 10.1073/pnas.0607015103 17085592 PMC1859931

[B59] Zentalla R Zhang Z-L Park M Thomas SG Endo A Murase K Fleet CM Jikumaru Y Nambara E Kamiya Y Sun T-P Global analysis of DELLA targets in early gibberellin signaling in *Arabidopsis* Plant Cell 2007 19 3037 3057 10.1105/tpc.107.054999 17933900 PMC2174696

[B60] Horsch RB Fry JE Hoffmann NL Eichholtz D Rogers SG Fraley RT A simple and general method for transferring genes into plants Science 1985 227 1229 1231 10.1126/science.227.4691.1229 17757866

[B61] Cutt JR Dixon DC Carr JP Klessig DF Isolation and nucleotide sequence analysis of cDNA clones for the pathogenesis-related proteins PR1a, PR1b, and PR1c of *Nicotiana tabacum *cv. *Xanthi *nc induced by TMV infections Nucleic Acids Res 1988 16 9861 10.1093/nar/16.20.9861 3186451 PMC338789

[B62] Pautot V Holzer FM Reisch B Walling LL Leucine aminopeptidase: An inducible component of the defense response in *Lycopersison esculentum *(tomato) Proc Natl Acad Sci USA 1993 90 9906 9910 10.1073/pnas.90.21.9906 8234334 PMC47681

[B63] Rozen S Skaletsky HJ Krawetz S, Misener S Primer3 on the WWW for general users and for biologist programmers Bioinformatics Methods and Protocols: Methods in Molecular Biology 2000 Totowa, NJ: Humana Press 365 386 10.1385/1-59259-192-2:365 10547847

[B64] Maurer-Stroh S Eisenhaber B Eisenhaber F N-terminal N-myristoylation of proteins: prediction of substrate proteins from amino acid sequence J Mol Biol 2002 317 541 547 10.1006/jmbi.2002.5426 11955008

